# Effects of Amyloid Precursor Protein 17 Peptide on the Protection of Diabetic Encephalopathy and Improvement of Glycol Metabolism in the Diabetic Rat

**DOI:** 10.1155/2013/689841

**Published:** 2013-06-17

**Authors:** Heng Meng, Duo Zhang, Haishan Yang

**Affiliations:** ^1^Department of Radiology, China-Japan Union Hospital of Jilin University, Changchun 130033, China; ^2^Department of Radiology, Affiliated Hospital of Beihua University, Jilin 132011, China

## Abstract

Researchers have proposed that amyloid precursor protein 17 peptide (APP17 peptide), an active fragment of amyloid precursor protein (APP) in the nervous system, has therapeutic effects on neurodegeneration. Diabetic encephalopathy (DE) is a neurological disease caused by diabetes. Here we use multiple experimental approaches to investigate the effect of APP17 peptide on changes in learning behavior and glycol metabolism in rats. It was found that rats with DE treated by APP17 peptide showed reversed behavioral alternation. The [^18^F]-FDG-PET images and other results all showed that the APP17 peptide could promote glucose metabolism in the brain of the DE rat model. Meanwhile, the insulin signaling was markedly increased as shown by increased phosphorylation of Akt and enhanced GLUT4 activation. Compared with the DE group, the activities of SOD, GSH-Px, and CAT in the rat hippocampal gyrus were increased, while MDA decreased markedly in the DE + APP17 peptide group. No amyloid plaques in the cortex and the hippocampus were detected in either group, indicating that the experimental animals in the current study were not suffering from Alzheimer's disease. These results indicate that APP17 peptide could be used to treat DE effectively.

## 1. Introduction

Diabetes mellitus, or simply diabetes, is a group of metabolic diseases in which a person has high blood sugar, either because the pancreas does not produce enough insulin or because cells do not respond to the insulin that is produced [1–3]. Insulin causes cells in the liver, skeletal muscles, and fat tissue to absorb glucose from the blood. In the liver and skeletal muscles, glucose is stored as glycogen, and in fat cells (adipocytes) it is stored as triglycerides [4, 5]. Diabetes can be divided into 3 main types: Type 1 diabetes, which results from the inability to produce insulin; Type 2 diabetes, resulting from insulin resistance; and gestational diabetes [6–8]. Type 2 diabetes, without proper treatment, can cause many complications, including hypoglycemia, diabetic ketoacidosis, and nonketotic hyperosmolar coma [9]. Adequate treatment of diabetes is consequently vital. Diabetic encephalopathy (DE) is caused by diabetes [10]. The complications of DE include memory loss, dementia, coma, seizures, and finally death. The defects in patients include lethargy, poor judgment and coordination of limbs, dementia, and muscle twitching [11].

Amyloid precursor protein (APP) is a transmembrane protein with six isoforms in the central nervous system (CNS), of which APP-695 is the most important [12, 13]. Hydrolysis of the N-terminus of APP-695 by *α*-secretase yields a soluble 100 kDa fragment, sAPP*α*, which has a unique function of promoting neuronal growth [14, 15]. The active domain responsible for this activity has been identified in the 319–335 peptide segment of APP-695 and is known as APP17 peptide. Previous studies reported that APP17 peptide could promote axonal growth, increase synaptic density, and protect neurons from ischemic damage [16, 17]. In a previous report from our group, we found that APP17 peptide ameliorates hippocampal neurodegeneration in mice afflicted by streptozotocin- (STZ-) induced diabetes [18]. The APP17 peptide has also showed neuroprotective effects through activation of specific signal transduction pathways associated with neuronal survival [19]. However, little is known about the effects of APP17 peptide on encephalopathy caused by diabetes mellitus. In the present study, we use multiple experimental approaches to investigate the effect of APP17 peptide on changes in learning behavior and glycometabolism in rats.

## 2. Materials and Methods

### 2.1. Experimental Animals and Creation of Animal Model

Male Wistar rats (weighing 180–200 g) were supplied by the Laboratory Animal Center of Beijing. All animal experiments were conducted according to the guidelines of the local animal use and care committees and executed according to the National Animal Law. The animals were divided into three groups: normal controls (CON, *n* = 25), diabetic (DE, *n* = 25), and APP17 peptide-protected group (DE + APP17 peptide, *n* = 25). APP17 peptide was synthesized by solid phase method and purified in the authors' laboratory. Rats in the DE + APP17 peptide group were given APP17 peptide for 4 weeks after STZ (Sigma) treatment (0.7 *μ*g per rat, s.c. daily). STZ was prepared before each use at 20 mg/mL in 0.1 M pH 4.4 citrate buffer and was injected at 150 mg/kg, i.p., into rats which had been fasted for 12 h prior to receiving the injection. Four days later, nonfasting blood glucose in a tail-vein sample was determined by a glucose analyzer; a value >15 mM/L was accepted as a successfully created diabetic model.

### 2.2. Learning and Memory

Morris water maze tests were performed after training for 12 weeks. After the rats were familiar with the testing environment, normal training was performed from the second day. Orientation test: rats were trained twice per day, one time in the morning and one time in the afternoon. Each training session lasted for 120 sec, and the gap time was 30 s. The training lasted for 4 days. The starting area was randomly selected, and the number of times rats touched the platform in 120 sec was recorded. The platform was removed, and the rats were placed into water at the opposite side of the platform. The percent of residence time in the center area and number of times of passing the former platform in 120 sec were recorded.

### 2.3. *In Vivo* Positron Emission Tomography (PET) Scans

PET studies were performed on the rats suffering from diabetes or DE (*n* = 20  per group). The PET protocol was the following: animals were anesthetized by isoflurane (3% for induction, 1%–1.5% for maintenance), and the respiration rate was monitored during the experiment. The body temperature of the animals was maintained at 37°C throughout the PET examination using a warming system.  PET images were recorded on a high-resolution small-animal PET imaging device with a spatial resolution of 1.35 mm and a field of view (FOV) of 7.6 cm (MicroPET Focus 220, Siemens Medical Solutions, Inc., Hoffman Estates, IL, USA) [20]. The mice were scanned with an energy window of 350–650 keV and a coincidence time window of 6 sec. Brain emission scans were acquired in 3D mode during 60 min after a tail-vein bolus injection MBq of [^18^F]-FDG (CisBio, Orsay, France).

Blood glucose concentration was measured once during the scan using a One Touch Ultra Glucose Meter (LifeScan, Issy-Les-Moulineaux, France). Blood glucose concentrations were in the normal range, and no difference was detected between the various genotypes or ages of the groups (2-way analysis of variance (ANOVA) with genotype and age as between-subject factors, *F*s (<1 for the main factors)). The PET images were reconstructed with the 2D iterative ordered-subset expectation maximization (FORE 2D OSEM) mode. Sixteen subsets and 4 iterations were used for reconstruction. The mean [^18^F]-FDG activities, corrected for radioactive decay, were evaluated for each VOI on integrated PET images recorded during a 30–60 min acquisition period. Standardized uptake values (SUVs) were obtained for each VOI by dividing the mean [^18^F]-FDG activities by the injected dose and the animal weight.

### 2.4. Biochemistry Markers

The brains of rats in each group after the test of abilities of learning and memory were collected on the ice, and then the hippocampus was dissected. Tissues were crushed and centrifuged at the speed of 2000 r/min for 10 min. The supernatant was collected, and the activities of SOD, GSH-Px, and CAT and content of MDA in the rat's hippocampal gyrus were investigated. Coomassie brilliant blue staining was used to detect protein concentration.

### 2.5. Harris Hematoxylin and Eosin (H&E) Staining

Thirty micron (*μ*m) brain coronal sections were collected from every 200 *μ*m section. The sections were deparaffinized, with two changes of xylene, 10 min each. The sections were rehydrated in 2 changes of absolute alcohol for 5 min each, 95% alcohol for 2 min, and 70% alcohol for 2 min, washed briefly in dH_2_O, and stained in Harris hematoxylin solution for 8 min. The sections were washed in running tap water for 5 min and differentiated in 1% acid alcohol for 30 sec. The slides were then washed in running tap water for 1 min and stained in 0.2% ammonia water or saturated lithium carbonate solution for 30 to 60 sec. The slides were then washed in running tap water for 5 min, rinsed (10 dips) in 95% alcohol, and counterstained in eosin-phloxine solution for 30 sec. The slides were dehydrated in 95% alcohol, 2 changes of absolute alcohol, 5 min each. The slides were cleaned in 2 changes of xylene, 5 min each, and mounted with xylene-based mounting medium. The neurons in CA1 in the hippocampus were observed using an optical microscope.

### 2.6. IHC Staining Test

After dissecting tissues at 5 *μ*m and fixed in 4% paraformaldehyde for 10 min, slides were incubated 2 to 3 times in xylene for 10 min each and then incubated twice in 100% ethanol for 2 min each. The slides were hydrated in 95%, 70%, 50%, and 30% ethanol for 2 min each. Slides were placed into buffer containing 5% normal goat serum for 10 min. Slides were incubated in a humidified chamber overnight with primary antibody (rabbit anti-rat Akt/PKB 1 : 500, rabbit anti-rat GLUT4 1 : 1000). They were washed in 5 m in buffer for 3 times and incubated with secondary antibody in a humidified chamber for 30 min. DAB and hematoxylin staining, 5 discontinue brain sections were selected, and 5 fields were selected randomly. The numbers of Akt/PKB and GLUT4 positive cells in CA1 were counted.

### 2.7. Cell Proliferation Assays

The inhibition of cell proliferation and viability of PC-12 cells was determined using the WST-1 (Roche) assay Kit. Cells were placed at 8,000 per well in 96-well plates in their respective growth medium with FBS reduced to 2%. The cells were allowed to grow for 24 h and then treated with different drugs. After 24 h, the WST-1 reagent was added to the plates according to manufacturer's protocol, and absorbance was read at 450 nm with an ELISA reader (Tecan). The results were used for calculating IC_50_ of APP17 peptide.

### 2.8. [^3^H]_2_-Deoxyglucose (2-DOG) Uptake Assay

PC-12 cells were placed at 8,000 per well in 96-well plates in their respective growth medium with FBS reduced to 2%. PC-12 cells were pretreated with Akt and PI-3K inhibitor 124005 (Millipore, USA) and Akt inhibitor 124011 (Millipore, USA). The cells were allowed to grow on 12-well plates and treated with APP17 peptide. The cells were serum-starved in DMEM containing 0.1% FBS for 3 h. The cells were washed twice with PBS and incubated in 0.45 mL of KRH (20 mM HEPES, pH 7.4, 136 mM NaCl, 4.7 mM KCl, 1.25 mM MgSO_4_, and 1.25 mM CaCl_2_) in the presence or absence of 10 nM insulin for 30 min at 37°C. For [^3^H] 2-DOG uptake, 50 L of reaction mixture containing 5 *μ*Ci of 2-[1,2-3H]-deoxy-D-glucose (PerkinElmer Life Sciences) and 1 mM 2-DOG was added to each well for 5 min at room temperature. The reaction was stopped by the addition of 50 *μ*L of 200 mM 2-DOG into each well. The cells were washed two times with ice-cold PBS and solubilized in 0.5 mL of 0.1% SDS at room temperature for 10 min. The cells were washed two times with ice-cold PBS and solubilized in 0.5 mL of 0.1% SDS at room temperature for 10 min. The incorporated radioactivity was determined by liquid scintillation counting of 400 *μ*L of each sample in triplicate. Nonspecific passive [^3^H] 2-DOG uptake control measured as a treatment with 10 *μ*M cytochalasin B was subtracted from each value.

### 2.9. Western Blot

Following heating at 100°C for 5 min, 20 *μ*g of protein was run. The *western blot *was run on an SDS-PAGE gel until the blue front was at the bottom of the gel. The gel was then transferred to a nitrocellulose membrane for 0.5 A. The membrane was blocked for 1 h in 5% skim milk in 1 × PBST. The membrane was incubated in the primary antibody (rabbit anti-rat p-Akt 1 : 500, rabbit anti-rat GLUT4 1 : 1000, rabbit anti-rat *β*-actin 1 : 200) at 4°C overnight. The membrane was then washed 3 times for 5 to 10 min in 50 mL of 1 × PBS with 0.1% Tween 20 at room temperature (RT). The membrane was incubated with goat anti-rabbit 1 : 200 for 1 h at RT in 1 × PBST, washed 3 × 10 min, and rinsed with dH_2_O. Detection of the protein was determined by the use of the ECL kit (2 mL/membrane). Briefly, in separate tubes, the black and white ECL solutions were mixed in a 1 : 1 ratio. The solution was then aliquoted onto the membranes and left standing for 1 min. The ECL was then drained off the membrane, and the membrane was wrapped in plastic and exposed to film. The expression of protein was compared with *β*-actin (a positive control).

### 2.10. Statistical Analysis

Data were expressed as mean ± standard deviation (M ± SD). Group differences in the swimming time in the Morris water maze test and the number of errors in the passageway water maze test were analyzed by SPSS 11.0 using Windows software to conduct two-way analysis of variance (ANOVA, equal variances assumed by S-N-K) on repeated measurements. Other data were analyzed by SPSS 11.0 using Windows software to conduct one-way ANOVA (equal variances assumed by S-N-K). A post hoc test was used to obtain the *P*  values. A  *P* < 0.05 was considered significant.

## 3. Results and Discussion

### 3.1. Memory Ability

**Figure 1:**
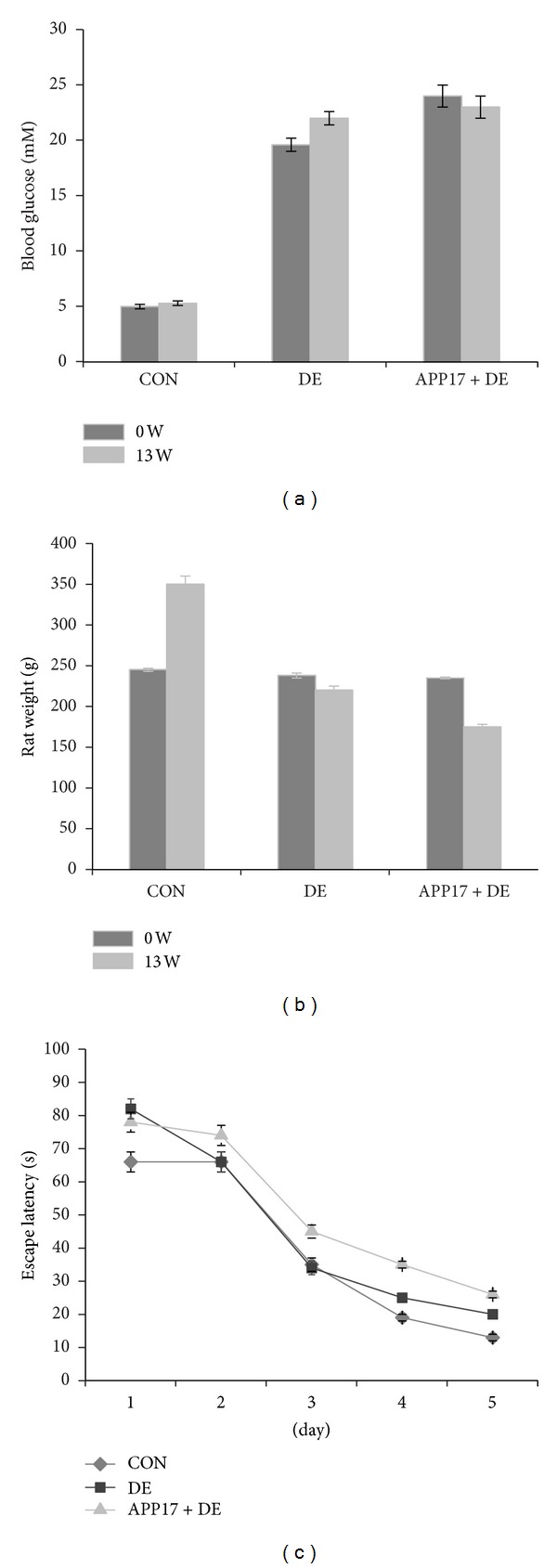
(a) Blood glucose and (b) body weight of mice in 3 groups. (c) The ability analysis of learning and memory of mice in 3 groups.

The rats of the DE group were polydipsia, polyphagia, polyuria and weight loss, yellowish color, poor spirit of the late, slow-moving symptoms. As shown in Table 1, at the beginning of generating animal model, the values of blood glucose in DE and DE + APP17 peptide groups were much higher than control group on 13th weeks (*P* < 0.01), while the body weight of mice in 3 groups remained the same (*P* > 0.05). After the treatment, the values of blood glucose in DE + APP17 peptide group were decreased, while body weight increased compared with DE group; the difference was significant (*P* < 0.05) (Figures 1(a) and 1(b)). Using the Morris water maze test, the rats treated with APP17 peptide had a prolonged swimming time (*P* < 0.05) and made significantly more errors when compared with the control group (*P* < 0.05). The rats showed reversed behavioral alternation with levels returning close to that of rats in the control group (Figure 1(c)).

### 3.2. The APP17 Peptide and Glucose Metabolism in the Brain of the DE Rat Model

As shown in Table 1, at the beginning of the generation of the animal model, the blood glucose in the DE and DE + APP17 peptide groups was significantly higher than that in the control group (*P* < 0.01), while the body weight of mice in 3 groups remained the same with no significant difference (*P* > 0.05). After the treatment, the blood glucose in the DE + APP17 peptide group decreased, and the body weight significantly increased compared with DE group (*P* < 0.05).

**Figure 2:**
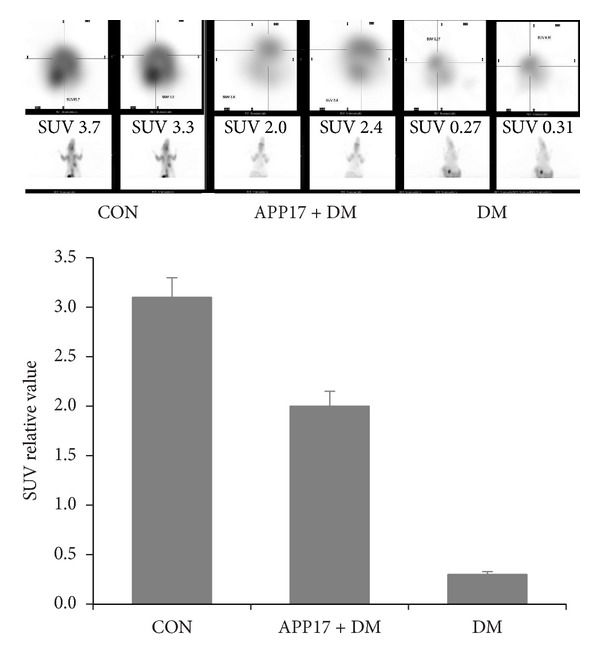
Evaluation of brain glycometabolism in DE animals by PET/CT.

The mean [^18^F]-FDG activities, corrected for radioactive decay, were evaluated for each VOI on integrated PET images recorded during a 30–60 minute acquisition period. Standardized uptake values (SUVs) were obtained for each VOI by dividing the mean [^18^F]-FDG activities by the injected dose and the animal weight. Regional FDG data were normalized by the FDG uptake within the cerebellum [21]. To study glycol metabolism, changes of [^18^F]-FDG-PET images were recorded in the DE rat model. After anatomofunctional combination, cerebral regions such as the cortex, the hippocampus, the striatum, and the cerebellum were outlined on PET images (Figure 2). A significant positive correlation was found between the DE group and DE + APP17 peptide group and the [^18^F]-FDG uptake in the cortex and the hippocampus. Evaluation of glycol metabolism in animals revealed a decrease of cortical and hippocampal glucose uptake in the DE group compared with the CON group. In the DE + APP17 peptide group, the glucose uptake was increased, as compared with DE group.

### 3.3. Biochemistry Alterations

Compared with the control group, the activities of SOD, GSH-Px, and CAT in the rat hippocampal gyrus in the DE group decreased significantly (*P* < 0.01). Compared with the DE group, the activities of SOD, GSH-Px, and CAT in the rat hippocampal gyrus were increased, whereas the MDA decreased significantly in the DE group (*P* < 0.05 or *P* < 0.01 (Table 2)).

### 3.4. Expression of Akt/PKB and GLUT4 after Treatment of APP17 Peptide

**Figure 3:**
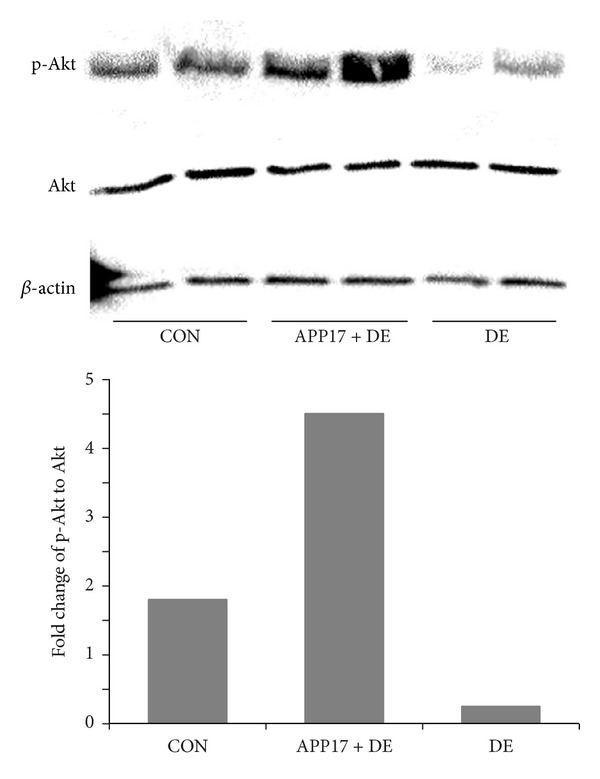
Enhanced insulin-induced Akt activation in the hippocampal gyrus of DE rats with APP17 treatment.

**Figure 4:**
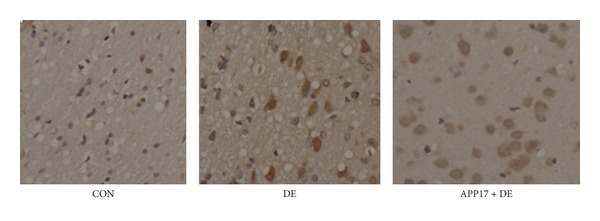
Enhanced GLUT4 expression in the hippocampal gyrus of DE rats with APP17 treatment.

The results of the IHC indicated that, compared with the control group, the number of Akt/PKB positive cells in the hippocampus of the DE group was reduced. In contrast the Akt/PKB positive cells in DE + APP17 peptide group was similar to those in the control group. The numbers of Akt/PKB positive cells in hippocampus in 3 groups are shown in Table 3. In the results of western blotting, APP17 acutely stimulated Akt phosphorylation in the group of treatment, compared with control cells (Figure 3). The expression of GLUT4 in membrane was obviously decreased in the rat hippocampal gyrus in DE group (*P* < 0.01). Compared with DE group, the expression of GLUT4 was obviously increased in the rat hippocampal gyrus in DE + APP17 group (*P* < 0.05) (Figure 4).

### 3.5. PI3K-Akt Pathway in Promoting Cell Glucose Metabolism by the APP17 Peptide

**Figure 5:**
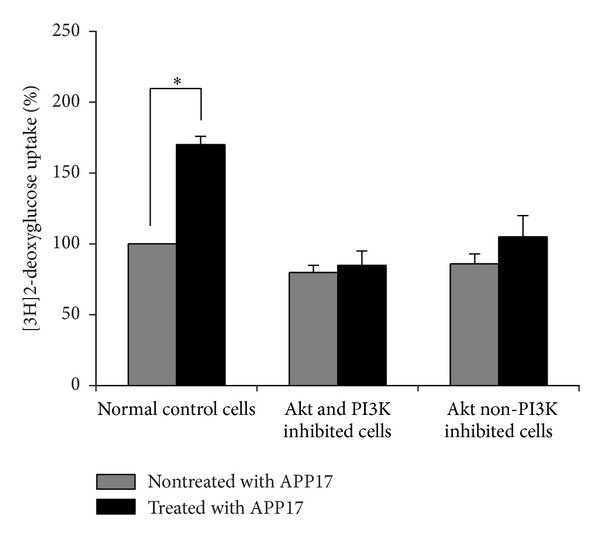
Enhanced insulin-induced glucose transport in PC-12 cells treated with APP17 peptide via PI3K-Akt pathway. Akt and PI3K inhibited cells pretreated with Akt inhibitor 124005; Akt non- PI3K inhibited cells were pretreated with Akt inhibitor 124011.

To identify the role of the PI3K-Akt pathway in the treatment of APP17 peptide, two specific inhibitors for the pathway were investigated in a glucose uptake assay *in vitro*. The Akt inhibitor 124011, a cell permeable and reversible benzimidazole compound, inhibits Akt phosphorylation/activation by targeting the ATP binding site of a kinase upstream of Akt but downstream of PI3K. Unlike phosphatidylinositol analog-based Akt inhibitors 124005, this inhibitor does not affect PI3K. PC-12 cells treated with the inhibitors or control were serum-starved for 24 h and then restimulated with insulin up to 20 h. The control cells and the PC-12 cells treated with the inhibitors were assayed for [^3^H]_2_-deoxy-D-glucose uptake after 16 h of insulin treatment. The group with the pretreatment of the inhibitors (Akt and PI3K inhibited cells and Akt non- PI3K inhibited cells) did not receive insulin stimulation. The incorporated radioactivity was determined by liquid scintillation counting of each sample in triplicate. Glucose uptake was not significantly altered in PC-12 cells treated with the inhibitors but was induced by 1.7-fold in normal control PC-12 cells (Figure 5).

All inhibitors that were used in the current study may affect both Akt1 and Akt2. According to Coleman's report, Akt2 plays an important role as a signaling molecule in the insulin signaling pathway. It is required to induce glucose transport. In a mouse model null for Akt1, but normal for Akt2, glucose homeostasis is unperturbed; however, the animals are smaller, consistent with a role for Akt1 in growth, and vice versa [22]. Hence it is inferred that APP17 peptide improves glucose metabolism in PC-12 cell via the Akt2-PI3K pathway.

No amyloid plaques in the cortex, nor the hippocampus, were detected in any of the groups. This indicates that the experimental animals in this study are not suffering from Alzheimer's disease, so the main reason affecting memory and learning ability is the diabetic encephalopathy.

At present, there are no effective drugs for the treatment of DE. This study showed that increased blood glucose levels were restored to normal in DE rats after treatment with APP17 peptide. This suggests that APP17 peptide acts by regulating glucose metabolism. The authors' laboratory has previously shown that APP17 peptide improves learning, memory function, and blood sugar concentration in STZ-induced DE rats and ameliorated the neurodegeneration of hippocampal neurons. This suggests that this peptide does not ameliorate DE through an unidentified action on a neuronal signal transduction pathway, rather through an insulinoid action. Previous reports have shown that cross-coupling between insulin and its receptors exists in human neuroblastoma cells [23].

These results show that glycometabolism plays an important role in the onset and development of neurodegenerative diseases and that the administration of APP17 peptide has neuroprotective effects against the changes induced by abnormal glycometabolism. APP17 peptide may cause these effects through the activation of common intracellular signaling pathways and initiation of “cross-talk” with neurotrophins. Further investigation is required to determine the mechanism by which APP17 peptide induces neuroprotection in this rat model. This may assist in identifying APP17 peptide as a potential therapeutic for neurodegenerative diseases.

## 4. Conclusions

The results of the current study indicate that APP17 peptide has a comprehensive therapeutic effect on diabetic encephalopathy, particularly through improving glycometabolism.

## Figures and Tables

**Table 1:** Blood glucose and body weight of mice in 3 groups (*x* ± *s*, *n* = 1). Different letters represent the significant difference at *P* < 0.05.

Group	Blood (mmol/L)		Body weight (g)	
	0 w	13 w	0 w	13 w
CON	5.40 ± 0.41	5.56 ± 0.35	240.87 ± 5.44	350.32 ± 19.19
DE + APP17	21.73 ± 1.53**	16.43 ± 1.12**	238.97 ± 5.91	250.58 ± 15.22**
DE	24.28 ± 1.98**	22.96 ± 1.35^∗∗##^	235.00 ± 12.1	180.02 ± 14.50^∗∗##^

*P < 0.05, **P < 0.01 versus CON, ^#^ *P* < 0.05, ^##^ *P* < 0.01 versus DE.

**Table 2:** Changes on biochemistry of rats in 3 groups (*x* ± *s*, *n* = 10).

Group	SOD (U/mg·pro)	GSH-Px (U/mg·pro)	CAT (U/mg·pro)	MDA (nmol/mg·pro)
CON	55.48 ± 5.22	0.072 ± 0.015	6.11 ± 0.80	8.01 ± 2.19
DE	44.87 ± 10.45^#^	0.050 ± 0.011^#^	2.42 ± 0.50^#^	15.32 ± 3.44^#^
APP17 + DE	60.50 ± 8.56^#^	0.062 ± 0.005^#^	3.98 ± 0.82^#^	10.15 ± 1.76^#^

^#^The significant difference at *P* < 0.05.

**Table 3:** The number of positive cells of neurons in hippocampus of rats in 3 groups (*x*±*s*).

Group	Akt/PKB positive cells	Live	Death
CON	35.56 ± 4.60	10	0
DE	18.75 ± 3.13^#^	7	3
APP17 + DE	31.68 ± 5.51	9	1

^#^Significant difference at *P* < 0.05.
